# Antioxidant Capacity of Macaronesian Traditional Medicinal Plants

**DOI:** 10.3390/molecules15042576

**Published:** 2010-04-12

**Authors:** Lucélia Tavares, Dina Carrilho, Meenu Tyagi, David Barata, Ana Teresa Serra, Catarina Maria Martins Duarte, Rui Oliveira Duarte, Rodrigo Pedro Feliciano, Maria Rosário Bronze, Paula Chicau, Maria Dalila Espírito-Santo, Ricardo Boavida Ferreira, Cláudia Nunes dos Santos

**Affiliations:** 1 Disease & Stress Biology, Instituto de Tecnologia Química e Biológica, Universidade Nova de Lisboa, 2781-901 Oeiras, Portugal; E-Mails: ltavares@itqb.unl.pt (L.T.); carrilho@itqb.unl.pt (D.C.); tyagi@itqb.unl.pt (M.T.); dbarata@gmail.com (D.B.); rbferreira@itqb.unl.pt (R.B.F.); 2 Nutraceuticals and Delivery Laboratory, Instituto de Tecnologia Química e Biológica/ IBET, Universidade Nova de Lisboa, 2781-901 Oeiras, Portugal; E-Mails: tserra@itqb.unl.pt (A.T.S.); cduarte@itqb.unl.pt (C.M.M.D.); 3 REQUIMTE, Faculdade de Ciências e Tecnologia, Universidade Nova de Lisboa, 2829-516 Caparica, Portugal; E-Mail: rod@dq.fct.unl.pt(R.O.D.); 4 Analytical Chemistry Laboratory, Instituto de Tecnologia Química e Biológica, Universidade Nova de Lisboa, 2781-901 Oeiras, Portugal; E-Mails: rodrigof@itqb.unl.pt (R.F.); mbronze@itqb.unl.pt (M.R.B.); 5 Faculdade de Farmácia da Universidade de Lisboa, Av. Prof. Gama Pinto,1649-003 Lisboa, Portugal; E-Mail: mrbronze@ff.ul.pt (M.R.B.); 6 Analytical Laboratory, Instituto de Tecnologia Química e Biológica, Universidade Nova de Lisboa, 2781-901 Oeiras, Portugal; E-Mail: chicau@itqb.unl.pt (P.C); 7 Instituto Superior de Agronomia, Centro de Botânica Aplicada à Agricultura, Universidade Técnica de Lisboa, Tapada da Ajuda, 1349-017 Lisboa, Portugal; E-Mails: dalilaesanto@isa.utl.pt (M.D.E.); rbferreira@isa.utl.pt (R.B.F.)

**Keywords:** antioxidant, L-ascorbic acid, selenomethionine, flavonoid, Macaronesia plants

## Abstract

The use of many traditional medicinal plants is often hampered by the absence of a proper biochemical characterization, essential to identify the bioactive compounds present. The leaves from five species endemic to the Macaronesian islands with recognized ethnobotanical applications were analysed: *Apollonias barbujana* (Cav.) Bornm., *Ocotea foetens* (Ainton) Baill, *Prunus azorica *(Mouill.) Rivas-Mart., Lousã, Fern. Prieto, E. Días, J.C. Costa & C. Aguiar, *Rumex maderensis* Lowe and *Plantago arborescens* Poir. subsp. *maderensis *(Dcne.) A. Hans. et Kunk.. Since oxidative stress is a common feature of most diseases traditionally treated by these plants, it is important to assess their antioxidant capacity and determine the molecules responsible for this capacity. In this study, the antioxidant capacity of these plants against two of the most important reactive species in human body (hydroxyl and peroxyl radicals) was determined. To trace the antioxidant origin total phenol and flavonoid contents as well as the polyphenolic profile and the amount of trace elements were determined. There was a wide variation among the species analysed in what concerns their total leaf phenol and flavonoid contents. From the High Performance Liquid Chromatography (HPLC) electrochemically detected peaks it was possible to attribute to flavonoids the antioxidant capacity detected in *A. barbujana*, *O. foetens, R. maderensis* and *P. azorica* extracts. These potential reactive flavonoids were identified for *A. barbujana*, *R. maderensis* and *P. azorica.* For *R. maderensis* a high content (7 mg g^-1^ dry weight) of L-ascorbic acid, an already described antioxidant phytomolecule, was found. A high content in selenomethionine (414.35 μg g^-1^ dry weight) was obtained for *P. arborescens* subsp. *maderensis* extract. This selenocompound is already described as a hydroxyl radical scavenger is reported in this work as also possessing peroxyl radical scavenging capacity. This work is a good illustration of different phytomolecules (flavonoids, organic acids and selenocompounds), presents in leaves of the five traditional medicinal plants endemic to Macaronesia, all exhibiting antioxidant properties.

## 1. Introduction

Oxidative stress represents a disturbance in the equilibrium status of prooxidant/antioxidant reactions in living organisms. The balance between antioxidants and damaging effects of free radicals is a very important aspect and is achieved by mechanisms collectively termed “redox regulation”, that protect living organisms from oxidative stresses [1]. The excess of reactive oxygen species (ROS) can damage cellular lipids, proteins or DNA, inhibiting their normal function [2]. Oxidative imbalance has been implicated in a number of human diseases as well as in the ageing process [2]. Therefore the study of ROS and antioxidants in biology triggered a medical revolution that underlies a new age of health and disease management [3]. The continuous upsurge of new challenges ranges from prevention of oxidative reactions in foods to novel pharmaceuticals for treatment/attenuation of chronic degenerative diseases, such as cancer, autoimmune, inflammatory, cardiovascular and neurodegenerative (e.g., Alzheimer's disease, Parkinson's disease, multiple sclerosis, Down’s syndrome) diseases, as well as ageing and cosmetics. 

Plant secondary metabolites, such as polyphenols, have been described as exhibiting important antioxidant, antimutagenic, anticarcinogenic, antiinflammatory and antimicrobial effects, that might be potentially beneficial in preventing diseases and contributing to human organism homeostasis.

Flavonoids are the largest group of polyphenols and over 2,000 individual flavonoids have been reported [4]. They are described as antioxidant molecules that scavenge free radicals with their concomitant oxidation and formation of a more stable free radical [5,6,7]. In recent years, there has been an increasing interest in investigating the various pharmacological properties of flavonoids [8]. 

The use of traditional medicine is widespread and the plants used constitute a large source of natural antioxidants that might serve as leads for the development of new drugs. The use of traditional herbs and medicinal plants has been traced to the occurrence of natural products and their health improvement capacities. However, other phytomolecules could act as antioxidant defences against ROS. Ascorbic acid (Vitamin C), Vitamin E, glutathione (GSH) and carotenoids are examples of non-enzymatic antioxidant defences [1]. There is also some elements, like Cu, Fe, Mn and Zn, and Se, that occur mostly bound to proteins, forming metalloenzymes that also possess important antioxidant activities. In humans, these trace elements accomplish decisive functions to maintain human health. Deficiency in any of them originates low levels of metalloproteins and/or low enzyme activities, leading to undesirable pathological conditions that may be readily prevented or reversed by adequate supplementation with the missing element. [9]

In this work, five endemic Macaronesian species, from the Azores and Madeira/Canary Islands, belonging to four different families and presenting a recognized ethnobotanical use or belonging to a genus with this sort of use [10,11,12,13,14,15,16,17,18,19] were selected (Table 1). Their contents in total phenols (TP) and total flavonoids (TF) were determined. Their antioxidant potential was assessed by measuring the antioxidant capacity for two of the most relevant free radicals for humans: peroxyl and hydroxyl radicals. For a better characterization of antioxidant capacity source, the polyphenolic profile and trace elements were assessed for all species. For *R. maderensis* additional quantification of L-ascorbic acid was performed and in the case of *P. arborescens* subsp. *maderensis, *free amino acid content was determined. Overall, for each plant under study, an attempt was made to relate their antioxidant capacity with the phytomolecules detected.

**Table 1:** Folk medicinal uses of *O. foetens*, *A. barbujana*, *R. maderensis*, *P. arborescens *subsp. *maderensis* and *P. azorica*.

Family	Plant name	Use in folk medicine
Lauraceae	*Ocotea foetens* (Ainton) Baill	*O. foetens* is used as antihypertensive, treatment for malignant diseases (poultices) and cancer [10,11]
	*Apollonias barbujana (Cav.) Bornm*	Plants of this family have been purported in folk medicine as diuretic, analgesic, antiulcerogenic, cytostatic, cardiotonic, expectorant, stomachic, sedative or carminative effects and against rheumatic pain [12]
Polygonaceae	*Rumex maderensis* Lowe	*R. maderensis* infusion is used as a diuretic and blood depurative and externally applied in poultices for dermatosis [11,13]* Rumex* sp. are used to treat headaches, to promote maturation abscess, in wound healing, for infected wounds and pimples [14,15]
Plantaginaceae	*Plantago arborescens* Poir*. *subsp. *maderensis* (Decne.) A. Hans. et Hunk.	*Plantago* sp. is used in infusion gargled to relieve sore throat, on the treatment of hepatitis, conjunctivitis, furunculosis, diarrhoea, malignant diseases, spasm, intestinal and stomach ulcers, stomach ache, tuberculosis, asthma, cough, bronchitis, boils, diabetes, goiter, it is also used as hemostatic, antitussive and expectorant and applied externally as a poultice to treat wounds, cuts and bee bites [10,11,15,16,17,18,19]
Rosaceae	*Prunus azorica* (Mouill.) Rivas Mart., Lousã, Fern.Prieto, E.Días, J.C.Costa & C.Aguiar	*Prunus* spp. are used in treatment of urinary tract diseases [16,18,19]

## 2. Results and Discussion

### 2.1. Total phenol and flavonoid content

**Figure 1:**
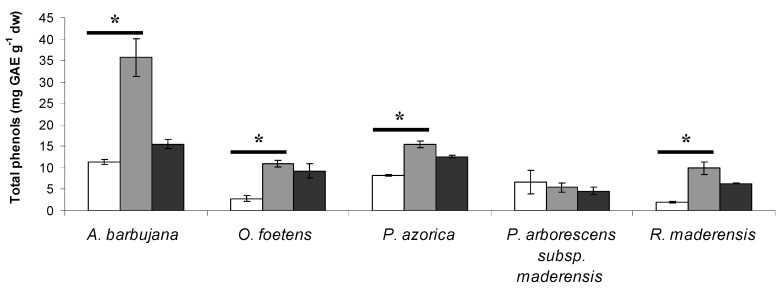
Total phenol content of the five Macaronesia plants under study. The extracts for the three types of solvents were evaluated for each plant: white bar- water; grey bar- water:ethanol (1:1); black bar- ethanol. Vertical bars represent ± SD. * - significantly different extractions at p < 0.05.

For each of the Macaronesia plants under study (*A. barbujana*, *O. foetens*, *P. azorica*, *P. arborescens *subsp*. maderensis* and *R. maderensis*), three leaf extractions with three clean solvents (water, 1:1 water-ethanol mixture and absolute ethanol) were used to select the best clean solvent for phenolic compound extraction (Figure 1). For all plants, the total content in polyphenols in the water-ethanol extract was either higher or not significantly different from the ethanolic extract. Subsequent HPLC analysis revealed that the water-ethanol extract possessed all peaks detected in the three types of extractions but in higher quantities (data not shown). For these reasons, further studies proceeded only for the water-ethanol extracts. *A. barbujana* presented the highest content in total phenolics in the water-ethanol extract (35.8 mg GAE g^-1^ dw, Table 2); all other species had values lower than 15.5 mg GAE g^-1^ dw. 

Since flavonoids are strongly associated with antioxidant capacity, the total flavonoid content in these plants was also determined (Table 2) for the water-ethanol extracts of plants studied. In accordance with phenol quantification, *A. barbujana* was the richest species in flavonoids, followed by *P. azorica*, *O. foetens*, *R. maderensis* and finally by *P. arborescens* subsp. *maderensis*. Based on these results, *P. azorica* had clearly a greater contribution of flavonoids to the total phenol content in contrast with *P. arborescens* subsp. *maderensis* which had a very low content in TF. There is no data available in the literature related with the phenolic and flavonoid leaf content of these species, despite their folk medicinal use, these plants were until now uncharacterized. 

**Table 2:** Total phenols (TP) content and total flavonoids (TF) content for the water:ethanol extracts of the five Macaronesia plants under study. Values are the mean of three independent replicates ± SD. Superscript letters are the significance levels at p < 0.05.

Plant species	Total phenols (TP) (mg GAE g^-1^ dw)	Total flavonoids (TF) (mg CE g^-1^ dw)
*A. barbujana*	35.8 ± 4.4 ^a^	18.31 ± 2.51 ^a^
*O. foetens*	10.9 ± 0.8 ^bc^	5.03 ± 0.42 ^c^
*P. azorica*	15.4 ± 0.8 ^b^	11.29 ± 0.21 ^b^
*P. arborescens * subsp. *maderensis*	5.4 ± 1.1 ^c^	0.22 ± 0.02 ^d^
*R. maderensis*	9.9 ± 1.6 ^bc^	5.23 ± 0.10 ^c^

### 2.2. Free radical scavenging activity

**Figure 2:**
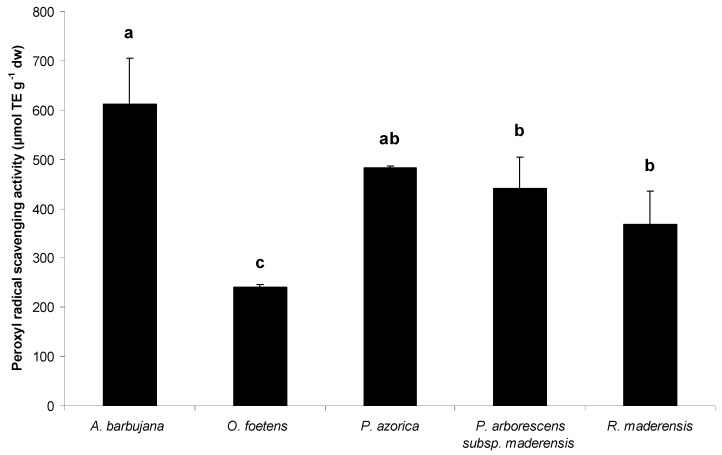
Peroxyl radical scavenging activity for the water:ethanol extracts of the five Macaronesia plants under study. Each point is the average of three independent replicates. Values are the mean of three independent replicates and bars represent SD. Superscript letters are the significance levels at p < 0.05.

**Figure 3:**
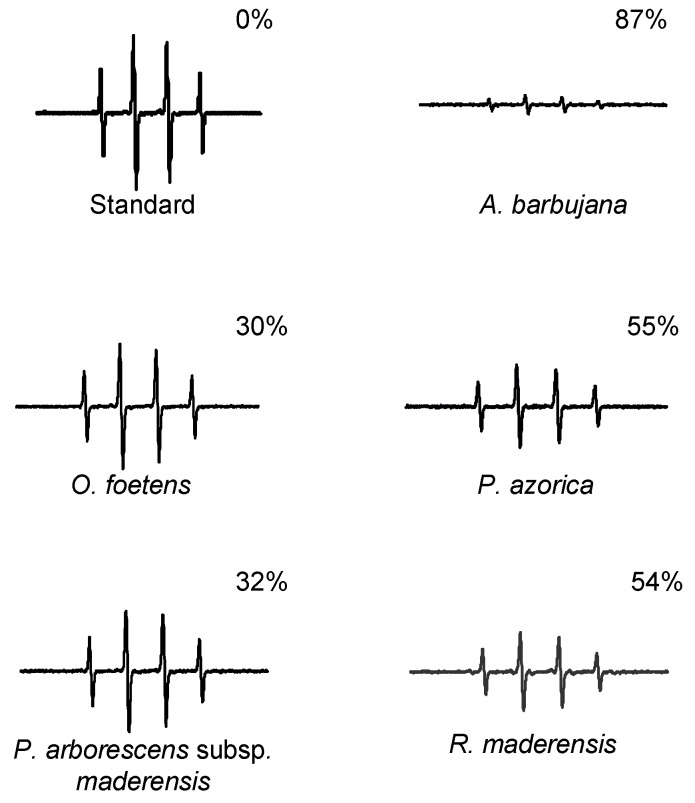
Effect of the water:ethanol extracts of the five Macaronesia plants under study on the EPR spectra, as described in the section 3.5. The percentage represents the relative attenuation of the EPR signal amplitude relative to that of the standard.

The capacity of plant extracts to scavenge peroxyl (ROO·) and hydroxyl (·OH) radicals was measured by the Oxygen Radical Absorbance Capacity (ORAC) method and Electron Paramagnetic Resonance (EPR), respectively (Figure 2 and Figure 3, respectively). These ROS are the two major free radicals with biological relevance in the human body. 

*A. barbujana* extract was clearly the most potent in scavenging both radicals (Figure 2 and Figure 3). In general, in plant extracts there is a positive relationship between TF (Table 2) content and antioxidant capacity, higher content in TF reflects in higher antioxidant capacity. However, some considerations should be discussed for some particular extracts. Concerning peroxyl radical scavenging capacity (Figure 2), it is interesting to consider the similar antioxidant capacity for *P. arborescens* subsp. *maderensis* and *P. azorica*, since they presented quite different TF content (Table 2). On the other hand, although *R. maderensis* and *O. foetens* presented similar TF content, the former exhibited twice the antioxidant capacity of the latter.

Similar analysis for hydroxyl radical scavenging capacity (Figure 3) led to equivalent conclusions. The magnitude of the difference in antioxidant capacity between *R. maderensis* and *O. foetens* remained the same (two fold). Curiously, *P. azorica*’s ability to scavenge hydroxyl radical was comparable to that of *R. maderensis*, although the former had a much higher TF content (Table 2). The same pattern is observed for *P. arborescens* subsp. *maderensis* and *O. foetens* – they presented a very different content in flavonoids, but similar hydroxyl radical scavenging capacity.

These results suggest either the existence of flavonoids displaying widely different antioxidant potencies and/or the possible occurrence of compounds interfering in antioxidant capacity and/or the presence of antioxidant molecules other than flavonoids. Previous works have already described the presence of interfering compounds in total extracts that after being removed allowed the enhancement of the extracts’ bioactivities [20,21]. To better understand the origin of antioxidant capacity from the leaf extracts of these plants, an additional number of experiments were performed and are discussed below.

### 2.3. HPLC profile

**Figure 4:**
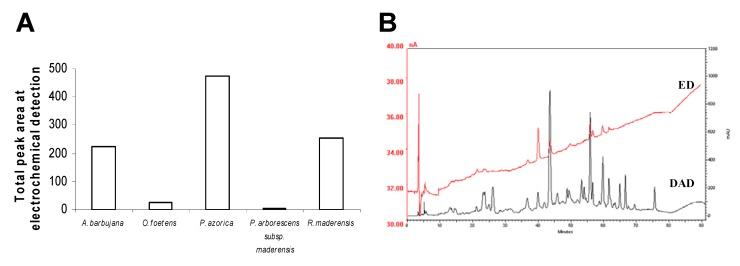

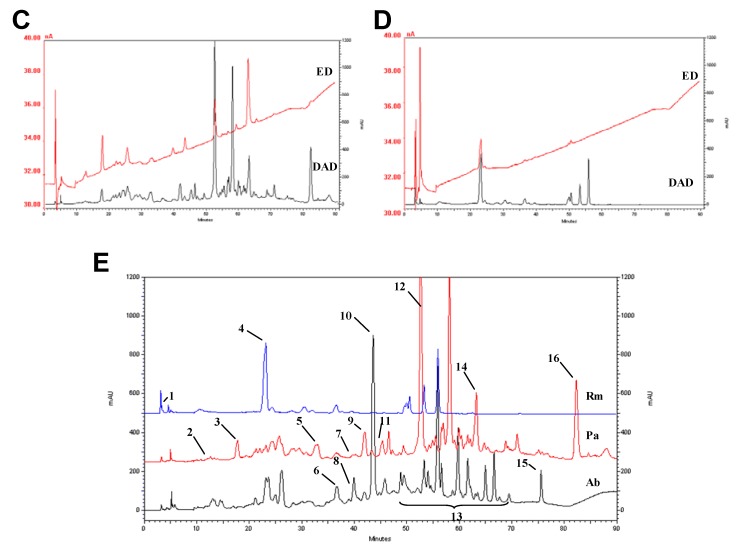
Phytochemical characterization of the five Macaronesia leaf water-ethanol extracts using the diode array (DAD) at 280 nm and the electrochemical detectors (ED) in tandem(-1 V, +1 V). (A) Total area of the peaks detected electrochemically for the five plant extracts studied; Chromatographic profiles of *A. barbujana *(B); *P. azorica *(C); *R. maderensis *(D); Chromatograms at 280 nm and peak identification (E) of *A. barbujana* (Ab), *P. azorica* (Pa) and *R. maderensis* (Rm). Peak identification: 1. Ascorbic acid; 2. Gallic acid; 3. Protocatechuic acid; 4. Neochlorogenic acid; 5. Caffeic acid; 6. Procyanidin B2; 7. Hydroxycinamic acid derivate; 8. Epicatechin; 9. Caffeic acid derivate 1; 10. Procyanidin; 11. Coumaric acid; 12. Rutin; 13. Quercetin/ kaempferol/ myrcetin glucosydes; 14. Caffeic acid derivate 2; 15. Apigenin; 16. Naringenin.

The compositional analysis of the water-ethanol leaf extracts from the plants under study was evaluated by High Performance Liquid Chromatography with Diode Array Detection and Electrochemical Detection (HPLC–DAD–ED). Peaks detected by the electrochemical detector correspond to reactive species with strong capacity to donate electrons. Therefore, a positive correlation between the total area of peaks detected in the electrochemical chromatogram (Figure 4A) and the antioxidant properties of the extracts (Figure 2 and Figure 3) is likely to be found. The sum of the area of the peaks detected in this system for each species is presented in Figure 4A. In this evaluation, *P. azorica* showed the greater total ED peak area, followed by *R. maderensis *and *A. barbujana*, with similar total areas; *O. foetens* and *P. arborescens *subsp. *maderensis* produced no significant areas. This evaluation reflects only those compounds that are distinguished by the electrochemical detector and that are involved in redox reactions.

*O. foetens* and *P. arborescens *subsp. *maderensis* originated HPLC profiles with several low intensity hardly detectable peaks (data not shown), as expected from their low flavonoid content (Table 2). The resulting chromatograms from the electrochemical detection and the UV spectroscopic detection at 280 nm are shown in Figure 4B to Figure 4D for *A. barbujana, P. azorica* and *R. maderensis*, respectively.

The total peak area obtained employing an electrochemical detector in the electrical detector for *P. azorica* may be explained by its higher content in TF (Table 2). This is in good agreement with the antioxidant properties detected by ORAC (Figure 3a) and EPR (Figure 3b). *A. barbujana*, that had shown the highest antioxidant potential for both radicals evaluated, had a total peak area from electrochemical detection equivalent to *R. maderensis*. The value plotted for *R. maderensis* in Figure 4A is mainly due to a single peak, as shown in the HPLC-ED profile (Figure 4D), identified as L-ascorbic acid (peak number 1, Figure 4E). This compound could contribute to the antioxidant capacities detected by ORAC and EPR [22,23,24]. The presence of ascorbic acid would explain the variation of the scavenging activity between *O. foetens* (devoid of L-ascorbic acid) and *R. maderensis*. *O. foetens* showed a lower antioxidant capacity than *R. maderensis*, however, both extracts presented similar amounts of polyphenols and/or flavonoids (Figure 3). The presence of L-ascorbic acid in *R. maderensis *was confirmed by an enzymatic method (9.00 ± 0.60 mg g^-1^ dw) and validated by an HPLC analytical methodology for organic acids, with the content in this compound determined as 7.0 ± 1.2 mg g^-1^ dw.

Tentative identification of the compounds fractionated by HPLC (Figure 4) has been made by examination of their UV spectra and comparison with standards. Each class of flavonoids has a typical UV absorption maxima and the classification proposed by Robards and Antolovich has been used in this work for identification purposes [25]. Flavones typically exhibit an intense band II (310–350 nm) absorption maximum with a shoulder or low intensity peak representing band I (250–280 nm). Flavonols absorb at 250–280 nm (band II) and 350–385 nm (band I), while hydroxycinnamic acids lack band I and exhibit absorption at 227–245 nm and at 310–332 (band II). 

Flavonoids identified in *A. barbujana* extract (Fig. 4B) that could be associated to its antioxidant capacity due to their redox reactivity (ED signals) were epicatechin, procyanidins and flavonol derivatives. In *P. azorica* extract (Figure 4CIn *R. maderensis* leaf extract, neochlorogenic acid was also found as contributing for antioxidant capacity in addition to the ascorbic acid contribution.

### 2.4. Trace elements and free amino acids

The results obtained above for the antioxidant properties of plant extracts suggest the presence of molecules other than polyphenols contributing to radical scavenging activity, this led us to determine selected trace elements contents by ICP (Inductively Coupled Plasma). Metals as Cu, Fe, Mn and Zn, and the non-metal Se, are considered trace elements because of their essentiality and their very limited requirements by humans. The biological activities of Cu, Fe, Mn and Se are strongly associated with the presence of unpaired electrons that allow their participation in redox reactions. Zn has no unpaired electrons but has been recognized to act as an antioxidant by replacing metals that are active in catalyzing free radical reactions [9].

The contents in the trace elements analyzed for *P. azorica* are in accordance with the values reported in the literature for other species of the same genus [26,27,28,29,30,31,32,33,34,35] (Table 3). There is no data available in the literature for these elements in plants of the genera *Ocotea* and *Apollonias*. Table 3 also shows, for *R. maderensis*, that while Cu and Fe contents are lower than the reported values, Zn content agrees with the literature. There is no information related to Mn and Se contents.

The discrepancies observed for plant species between the trace element content reported in Table 3 in comparison with values in literature may be due to species specific differences or to differences in the amount and form in which these elements are available to the plants in the soil [36]. 

**Table 3:** Contents in Cu, Fe, Mn, Se and Zn of the five Macaronesia plants under study. The values reported here from the literature refer to species that belong to the same genus.

Plant species	Cu (µg g^-1^ dw)		Fe (µg g^-1^ dw)		Mn (µg g^-1^ dw)		Se (µg g^-1^ dw)		Zn (µg g^-1^ dw)	
	Determined values	Reference values	Determined values	Reference values	Determined values	Reference values	Determined values	Reference values	Determined values	Reference values
*A. barbujana*	4.09		59.91		63.03		0.07		14.26	
*O. foetens*	12.43		34.40		12.34		0.13		14.47	
*P. azorica*	6.87	4.8-23.8 ^a^^,b,g,h^	66.81	25.18-1117 ^h,i,j,k^	16.65	1.8-74.8 ^a^^ ,b,g,h^	0.07	0.003-0.088 ^g^^,h^	11.99	1.8-49.8 ^a^^ ,b,g,h^
*P. arborescens * subsp.* maderensis*	13.58	15.2-41.1 ^e^	160.64		27.17	80.5-229 ^e^	0.26	0.2 ^f^	46.08	36.5-71.8 ^e^
*R. maderensis*	9.75	19-39 ^c,d^	190.68	241-425 ^h^	29.20		0.13		47.71	37-120 ^c,d^

Legend: a [26]; b [27]; c [28,29]; d [30]; e [31]; f [32]; g [33]; h [35]; i [34]; j [33]; k [29].

For *P. arborescens *subsp*. maderensis*, Cu and Mn contents are lower than those already described, while the Se content (0.26 μg g^-1^ dw) is above the expected value for *Plantago* genus. Plants convert Se mainly into selenomethionine, about 50% of the total Se content, whereas selenocysteine (Se-Cys), methyl-Se-Cys and g-glutamyl-Se-methyl-Cys are not significantly incorporated into plant protein and are at relatively low levels irrespective of soil Se content [37]. Selenomethionine has been described as a potent antioxidant molecule [38,39,40,41], on the other hand higher animals are unable to synthesize it and the ingested Se-Met is absorbed in the small intestine by active transport in mammals [37].These evidences make this molecule very interesting antioxidant with biological significance. The presence of high contents in this trace element in the *P. arborescens *subsp*. maderensis* extract, may contribute to its antioxidant properties, since this plant contains scarce detectable flavonoids, or other relevant compounds detected by HPLC- DAD (results not shown).

In order to confirm the presence of a biological active antioxidant species, total free amino acid profile of *P. arborescens* subsp. *maderensis* leaf extract was obtained by HPLC-DAD as described in Section 3.9. The results of the free amino acid quantification, presented in Table 4, illustrate the presence of high selenomethionine and selenocysteine contents in the leaves of *P. arborescens* subsp. *maderensis*, when compared with other free amino acids. 

**Table 4:** Contents of free amino acids present in the water:ethanol extract of *P. arborescens *subsp. *maderensis.*

Free amino acid	Molecular weight	Content (μg g^-1^ dw)
Hydroxyproline	131.1	253.60
Serine	105.1	23.11
Asparagine	132.1	83.35
Proline	115.4	105.40
Se-Cysteine	182.08	122.60
Valine	117.2	152.72
Se-Methionine	196.11	414.35

At concentrations present in *P. arborescens* subsp. *maderensis* extract (Table 5), selenomethionine possessed an antioxidant capacity against peroxyl radical equivalent to 12.90 TE, while in the same conditions selenocysteine presents 0.63 TE. This is the first time that is reported peroxyl radical scavenging capacity exhibited by selenomethione. This compound has already being referred in literature as displaying antioxidant capacity for free radicals such as hydroxyl and peroxynitrite [39,40,41]. These results point out that selenocompounds, in particular selemomethionine are contributing for the antioxidant capacity of *P. arborescens *subsp. *maderensis* leaf extract, practically devoid of flavonoid compounds. 

**Table 5:** Antioxidant capacity of pure selenoamino acids commercial available at the concentrations present in *P. arborescens* subsp. *maderensis* extract. Values are the mean of three independent replicates ± SD.

Free amino acid	Concentration (mg mL^-1^)	Peroxyl radical scavenging activity (TE)
Se-Cysteine	0.0024	0.63 ± 0.13
Se-Methionine	0.0080	12.90 ± 2.15

Selenium supplementation has already been described as reinforcing the endogenous antioxidative systems and therefore being beneficial as an adjuvant therapy for some human pathologies. Selemethionine is efficaciously absorbed and retained in animals and presents antioxidant properties for biologically significant free radicals, as discussed. In this work *P. arborescens *subsp. *maderensis *revealed as an interesting source of this physiologically relevant selenocompound.

## 3. Experimental

### 3.1. Plant material

Leaves from five plants endemic to the Macaronesian islands were collected in December 2006 at the Ajuda Botanical Garden, Lisbon, Portugal, and stored at -80 ºC. The selected plants were *Apollonias barbujana* (Cav.) Bornm. (LISI 557/2009, M.D. Espírito Santo), *Ocotea foetens *(Aiton) Baill (LISI 558/2009, M.D. Espírito Santo), *Prunus azorica* (Mouill.) Rivas-Mart., Lousã, Fern. Prieto, E. Días, J.C. Costa & C. Aguiar (LISI 562/2009, M.D. Espírito Santo), *Rumex maderensis* Lowe (LISI 563/2009, M.D. Espírito Santo), and *Plantago arborescens* Poir. subsp. *maderensis *(Dcne.) A. Hans. et Kunk. (LISI 559/2009, M.D. Espírito Santo). For all species, voucher samples were authenticated and deposited at the herbarium "João de Carvalho e Vasconcelos", Instituto Superior de Agronomia, Lisbon, Portugal.

### 3.2. Extraction procedure

For each plant species, three different extractions were performed using three clean solvents (water, 1:1 water-ethanol mixture, and absolute ethanol) to select the best clean solvent for the extraction of phenolic compounds. Plant leaves were ground to a fine powder with a grinder. To each 1 g of powder, solvent (6 mL) was added and the mixture was shaken for 30 min, at room temperature in the dark. The mixture was then centrifuged at 12,400 *g*, during 10 min at room temperature. The supernatant was filtered through paper filter and then through 0.20 µm cellulose acetate membrane filters. The resulting extracts were frozen at -80 ºC under a nitrogen atmosphere and subsequently dried under vacuum to determine the dried extract yield. The yields of dried extracts were 11% (w/w) of the starting material for *A. barbujana *and *P. azorica*, and 5% (w/w), 4% (w/w) and 3% (w/w) for *O. foetens*, *P. arborescens *subsp. *maderensis* and *R. maderensis*, respectively. 

### 3.3. Total phenolic content

Determination of total phenolic compounds was performed by the Folin-Ciocalteau method [42]. Briefly, to each well of a microplate water (235 µL), sample (or solvent, in the control, 5 µL), Folin-Ciocalteau’s reagent (15 µL, Fluka®) and saturated Na_2_CO_3_ aqueous solution (45 µL) were added. The microplate was incubated for 30 min at 40 ºC and the absorbance at 765 nm measured. Gallic acid was used as the standard and the results are expressed in mg of gallic acid equivalents per gram of dry weight (mg GAE g^-1^ dw) of plant material. 

### 3.4. Total flavonoid content

Measurement of flavonoid content was performed by a modification of the AlCl_3_ complexation method [43]. To each well of a 96-well plate, water (125 µL), sample (or solvent, in the control, 25 µL) and 5% (w/v) NaNO_2_ (7.5 µL) were added. The plate was incubated for 6 min at room temperature and then 10% (w/v) AlCl_3 _ (15 µL) were added. After incubation during 5 min, 1 M NaOH (100 µL) was added and the solution of each well was mixed. The absorbance was measured at 510 nm. (+)-Catechin hydrate, minimum 98% (w/w) was used as the standard, and the results are expressed as mg catechin equivalents per gram of dry weight (mg CE g^-1^ dw) of plant material. 

### 3.5. Free radical scavenging assays

#### 3.5.1. Peroxyl radical scavenging capacity

Peroxyl radical scavenging capacity was determined by the ORAC method [24,44]. Briefly, the reaction mixture contained sodium fluorescein (1,800 µL, 4 nM, Uranine, Fluorescein Sodium Salt®, TCI Europe), sample (300 µL) and 2,2’-azobis(2-amidopropane)dihydrochloride (300 µL, 41.4 g L^-1^). The blank contained 75 mM phosphate buffer (pH 7.4, 300 µL) instead of the sample, whereas for the calibration it contained of 5 to 50 µM 6-hydroxy-2,5,7,8-tetramethylchroman-2-carboxylic acid (Trolox®, 300 µL). The decrease of fluorescence was monitored kinetically during 30 min at 37 ºC (excitation: 493 nm; emission 515 nm) on a Varian Eclipse 96 Well spectrofluorimeter. The final results were calculated by the difference of area under the curve of fluorescence’s decay between blank and sample and are expressed as µmol Trolox® equivalents per gram of dry weight (µmol TE g^-1^ dw) of plant material.

#### 3.5.2. Hydroxyl radical scavenging assay

The ability of the plant extracts to scavenge the hydroxyl radicals in the presence of hydrogen peroxide was evaluated by electron paramagnetic resonance (EPR) with the use of the spin-trapping agent, 5,5-dimethyl-1-pyrroline-*N*-oxide (DMPO), as described previously [45]. All EPR measurements were conducted using a Bruker EMX6/1 spectrometer (Bruker Instruments, Billerica, MA, USA) and a flat cell assembly. The EPR spectrometer settings were: receiver gain, 4.48 × 10^-4^; time constant, 20.48 s; modulation amplitude, 1.0 G; scan time, 83 s; and magnetic field, 3450 ± 100 G, with an X-band frequency of 9.69 GHz. 

Reactants were mixed in test tubes, containing a phosphate-buffered solution (pH 7.4) with 10 mM DMPO, 0.4 mM FeSO_4_, 2 mM H_2_O_2_ and plant extract (100 μL) in a final volume of 500 μL. The reaction mixture was then transferred into a flat cell and the EPR spectra recorded 3 min after reaction initiation. Experiments were performed at room temperature and under ambient air. The signal intensity of DMPO-OH radical adduct was obtained by double integration and the scavenging activity of the extract was estimated as the percent decrease of the relative intensity of the signal with reference to that of the control (without extract).

### 3.6. HPLC profile of phenolic compounds

HPLC analysis was performed as described earlier [46], with a Thermo Finnigan Surveyor instrument (San Jose, CA, USA) equipped with a diode array detector (Thermo Finnigan—Surveyor,) and an electrochemical detector (Dionex, ED40). Briefly, separations were performed at 35 ºC on a LiChrospher C18 (5 μm, 250 mm × 4 mm i.d.) column (Merck) with a guard column of the same type. The samples extracted as described above were injected using a 20 μL loop. The separations were carried out with a flow rate of 700 μL min^-1^ and the mobile phase consisted of a gradient mixture of eluent A (phosphoric acid, 0.1%, v/v) and eluent B (phosphoric acid–acetonitrile–water 5:400:595, v/v/v). The following gradient of eluents was used: 0–15 min from 0 until 20% eluent B; 10 min with 20% eluent B; 25–70 min, from 20 until 70% eluent B; 70–75 min, with 70% eluent B; 75–85 min from 70 until 100% eluent B; 85–90 min, with 100% eluent B. 

Diode array detection was performed between 200 and 800 nm. Electrochemical detection was programmed for a linear variation from −1.0 V to 1.0 V in 1.00 s (detection by integrated voltammetry using a cyclic variation of the potential). The measurements were taken with a 50 Hz frequency with an analogic/digital converter. The data acquisition systems were the Chromquest version 4.0 (Thermo Finnigan—Surveyor) for the diode array detector and the Unicam 4880 software for the electrochemical detector.

### 3.7. Quantification of L-ascorbic acid

L-Ascorbic acid content in the extract previously prepared was quantified by an enzymatic method, using the kit K-asco from Megazyme, according to the manufacturer instructions. To validate the L-ascorbic acid quantification it was performed a new plant extraction using phosphoric acid (6 mL g^-1^ fw). The homogenate was then centrifuged at 13,000 *g* for 20 min at 4 ºC and the supernatant filtered using 0.20 µm cellulose acetate membrane filters. L-Ascorbic acid was determined using an isocratic method with glacial acetic acid 5% (v/v) and a flow rate of 1 mL min^-1^. The oven temperature was set to 25 ºC, and 20 μL sample extract were injected into the Thermo Finnigan HPLC with detection wavelength set at 262 nm. A LiChrospher C18 (5 μm, 250 mm × 4 mm i.d.) column (Merck) and a guard column of the same packing material were used. Washing of the column system was performed with 80% (v/v) acetonitrile and 20 % (v/v) water during 40 min. After washing, column was stabilized with the mobile phase. Standard solutions of L-ascorbic acid prepared in water with concentration values between 10 and 200 mg L^-1^ were prepared. A linear regression curve was obtained (R^2 ^= 0.9995). 

### 3.8. Mineral composition

Plants were collected, washed with water and dried to a constant weight. The samples were then acid-digested with concentrated nitric acid and the minerals determined by Inductively Coupled Plasma-Optical Emission Spectrometry (ICP-OES), except for Se which was determined by Inductively Coupled Plasma- Mass Spectroscopy (ICP-MS; LMI, AB Lennart Mansson International).

### 3.9. HPLC quantification of free amino acids

Extraction was done by adding 12 volumes of 50% (v/v) ethanol for each gram of dry leaf powder. The samples were stirred for 30 min and centrifuged (3,500 g, 30 min). The pellets were washed twice with 50% (v/v) ethanol. The supernatants were collected, concentrated under vacuum and finally stored at -20 ºC. The free amino acid content of the extracts were analysed on a HPLC (Alliance Waters 2695, with a PDA Detector, 2996) gradient system with precolumn phenyl isothiocyanate (PITC) derivatization of the samples with a NovaPack C18 (4 µm, 3.9 mm × 300 mm i.d.) column, as described previously [47]. Data acquisition and analysis were made by Empower Software (Waters, Empower Pro, 2002).

### 3.10. Statistical analysis

The results reported in this work are the average of at least three biological replicates and are represented as the mean ± SD. Differences among treatments were assessed by analysis of variance [48] with Tukey HSD (Honest Significant Difference) multiple comparison test (α = 0.05) using SigmaStat 3.10 (Systat).

## 4. Conclusions

The Plant Kingdom represents a wide reservoir of phytocomplexes and natural compounds that still wait for a proper identification and characterization. Among them, many have recognized ethnopharmacological relevance and are “heavy loaded” with bioactive compounds.

The five selected species (*A. barbujana*, *O. foetens*, *P. azorica, P. arborescens* subsp. *maderensis *and *R. maderensis)*, all endemic to Macaronesia, are used in folk medicine to treat diseases in which oxidative stress seems to be involved. Thus, it is crucial to determine not only their antioxidant capacity but also to characterize its origin. For *O. foetens, P. azorica, *and *A. barbujana* the antioxidant capacity seems to be associated mainly to their flavonoid contents. For *R. maderensis *and *P. arborescens* subsp. *maderensis*, other phytomolecules than flavonoids, were found to contribute to their antioxidant capacity. Ascorbic acid was related to *R. maderensis *antioxidant capacity, while selenomethionine was related with *P. arborescens* subsp. *maderensis *antioxidant capacity*. *This selenocompound, already described with hydroxyl radical scavenging capacity, was also reported in this work as presenting peroxyl radical scavenging capacity. In conclusion, this study shows that chemically distinct groups of compounds (flavonoids, organic acids and selenocompounds) contribute differentially for antioxidant properties of the five medicinal plants endemic to Macaronesia.
